# Supplementary material for: Personalized deep neural networks reveal mechanisms of math learning disabilities in children
Source: Sci Adv. 2025 Jun 6;11(23):eadq9990. doi: 10.1126/sciadv.adq9990 (PMC13109960; doi:10.1126/sciadv.adq9990)
Supplement: Supplementary file 1 — Supplementary Text Figs. S1 to S9 Table S1 References [file sciadv.adq9990_sm.pdf]

Science Advances
AAAS

Supplementary Materials for

Personalized deep neural networks reveal mechanisms of math learning disabilities in children

Anthony Strock *et al.*

Corresponding author: Anthony Strock, astrock@stanford.edu; Vinod Menon, menon@stanford.edu

Sci. Adv. **11**, eadq9990 (2025)
DOI: 10.1126/sciadv.adq9990

This PDF file includes:

Supplementary Text
Figs. S1 to S9
Table S1
References

Supplementary Text

pDNN model architecture

The pDNN model is based on the CORNet-S architecture (Kubilius et al., 2018) (*14*). First, V1 receives as input a 28x140 picture with 3 color channels, that is, a 3x28x140 tensor. V1 transforms this input into a 6x7x35 tensor through a chain of a 7x7 convolution with stride 2 augmenting the number of channels to 64, a 3x3 max pooling with stride 2, and a 3x3 convolution, each of the convolution being followed by batch normalization and a ReLU non-linearity. The output of V1 (V2, V3) is then fed as input to V2 (V3, IPS), which transforms it into a 128x4x18 (resp. 256x2x9, 512x1x5) tensor. V2, V3, and IPS are built with the same building block that resembles a recurrent version of a residual block of the ResNet architecture, which has proven to be one of the best performing models on various benchmark datasets in different domains (He, Zhang, Ren, & Sun, 2016) (*74*). This building block is recurrent, but only the last output it produces is being fed as input to the next block. At time $t = 0$, the input to the block is the last output produced by the previous block, which is first transformed through a 1x1 convolution increasing two times its number of channels. For the following time steps, the input to the block is directly replaced by the feedback of its own output at the previous time step. Moreover, at each time step, the block transforms its input through a chain of 3 convolutions, each followed by batch normalization and a ReLU non-linearity: (1) a 1x1 convolution increasing four times the number of channels, (2) a 3x3 convolution being performed with stride 2 at time $t = 0$, and with stride 1 at all other times, and (3) a 1x1 convolution decreasing four times the number of channels. Furthermore, a skip connection is added between the input before the last ReLU non-linearity which, at time $t = 0$, adds a 1x1 convolution of the input with stride 2 before passing it through the nonlinearity, and at all other times, simply adds the input of the block before passing it through the nonlinearity. V2 and IPS are running for 2 timesteps, whereas V3 is running for 4 timesteps. We focus the analysis of neurons in V2, V3 and IPS on their last timestep. Finally, to produce the output of the whole model, the last output from IPS is fed in a simple linear decoder preceded by an adaptative average pool which enforces the input of this linear decoder to be of dimension 512 (i.e., the number of output channels of IPS). In the original work, the output has 1000 dimensions as there are 1000 different classes in ImageNet. In this work, we only changed the output dimension of that last linear decoder to match the number of classes we consider, that is, 19 dimensions for 19 classes representing each of the 19 different results from 0 to 18 we consider. Code for CORNet-S can be found at: https://github.com/dicarlolab/CORnet.

Statistical Analysis

We conducted statistical analyses using NumPy/SciPy. We compared group means with independent t-tests and quantified effect sizes using Cohen’s d. We assessed relationships between variables with Pearson’s correlation. As per *(20)*, we excluded one participant (out of the initial 46) due to an invalid *NumOps* subtest score caused by administrator error.

pDNN analysis using printed numerical stimuli

In real-world educational environments, children learn to process numbers presented in various formats - handwritten, printed, and digital. To better reflect this ecological context, we expanded our analyses to use a training set that combines both handwritten and printed numerical stimuli. Results demonstrate that our findings about the role of neural excitability in MLD are robust across different types of numerical stimuli (**Fig. S8-9**). We observed the same pattern of slower learning rates with higher neural excitability, and successful behavioral matching of MLD and TD participants to high and low excitability models, respectively. The analysis confirmed higher addition-subtraction neural representational similarity in MLD pDNN IPS, with a significant correlation between model and child IPS neural patterns (r = 0.58). MLD pDNNs continued to show greater numerical systematic error, reduced response diversity, and higher inter-manifold correlations in the IPS. Importantly, the finding that additional training can lead to behavioral improvements in MLD pDNNs was also replicated.

These results demonstrate that our findings about the role of neural excitability in mathematical learning disabilities are robust across different types of numerical stimuli.

Neural representational similarity across overlapping operands

To investigate how neural excitability affects the representation of problems with varying degrees of operand overlap, we analyzed NRS between four categories of problem pairs:

1. Problems sharing both operands (e.g., "6+2" vs "6-2")
2. Problems sharing only the left operand (e.g., "6+2" vs "6-3")
3. Problems sharing only the right operand (e.g., "5+2" vs "6+2")
4. Problems with no shared operands (e.g., "5+2" vs "6-3")

In the IPS layer, increasing neural excitability was associated with higher NRS across all categories (**Fig. S2**). Strong positive correlations were observed between neural gain and NRS for problems with both operands shared (r = 0.89, p < 0.001), left operand shared (r = 0.95, p < 0.001), right operand shared (r = 0.96, p < 0.001), and no operands shared (r = 0.97, p < 0.001).

At low levels of neural excitability (G = 1), problems sharing both operands showed higher NRS compared to problems with no shared operands (pairwise difference between shared operands and no shared operands: $M = -0.21, SD = 0.33, t = 5.19,\ p < 10^{-4}$). This difference progressively diminished with increasing neural excitability. Specifically, pairwise difference between NRS of problems sharing both operands and NRS of problems with no shared operands is higher for G=1 ($M = 0.21, SD = 0.07$) than for G=5 ($M = 0.07, SD = 0.10$) ($t = 2111.03,\ p < 10^{-323}$)

These findings suggest that while low neural excitability allows the network to maintain some differentiation between related and unrelated problems, higher excitability leads to a general deficit in forming distinct neural representations regardless of problem similarity. This pattern indicates that neural hyper-excitability impairs the network's ability to form

precise and differentiated representations of numerical problems, extending beyond mere confusion between highly related problems.

This analysis provides additional support for our main finding that neural hyper-excitability fundamentally disrupts the formation of distinct neural representations for different numerical problems, potentially contributing to the broader mathematical difficulties observed in children with MLD.

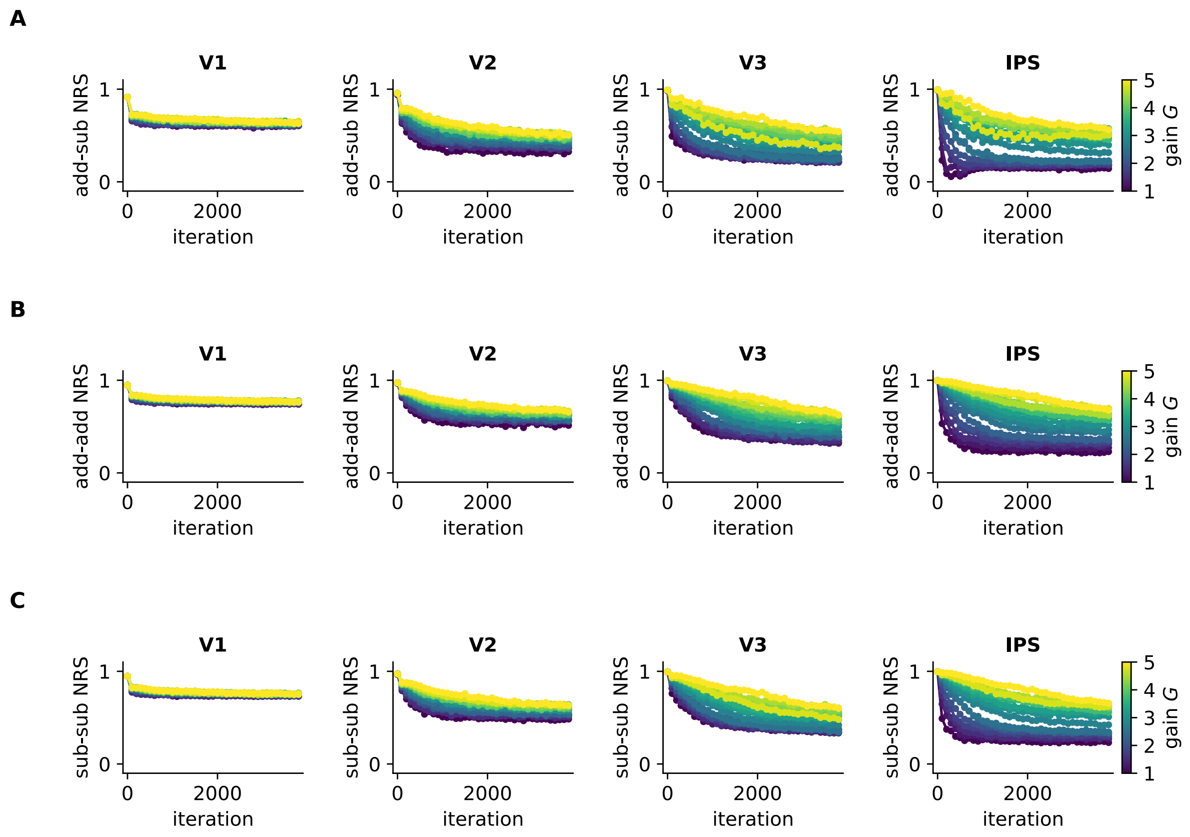

Fig. S1. Hyper-excitability diminishes differentiations of neural representations across iterations and across pDNN layers.

Evolution of DNN NRS for different levels of neural excitability measured as neural gain G. Neural gain G is represented by color, varying from blue ($G = 1$) to yellow ($G = 5$). **A-C.** As neural excitability increases, **A.** NRS between addition and subtraction problems (add-sub NRS), **B.** NRS between addition problems (add-add NRS), and **C.** NRS between subtraction problems (sub-sub NRS) show slower decreases with training.

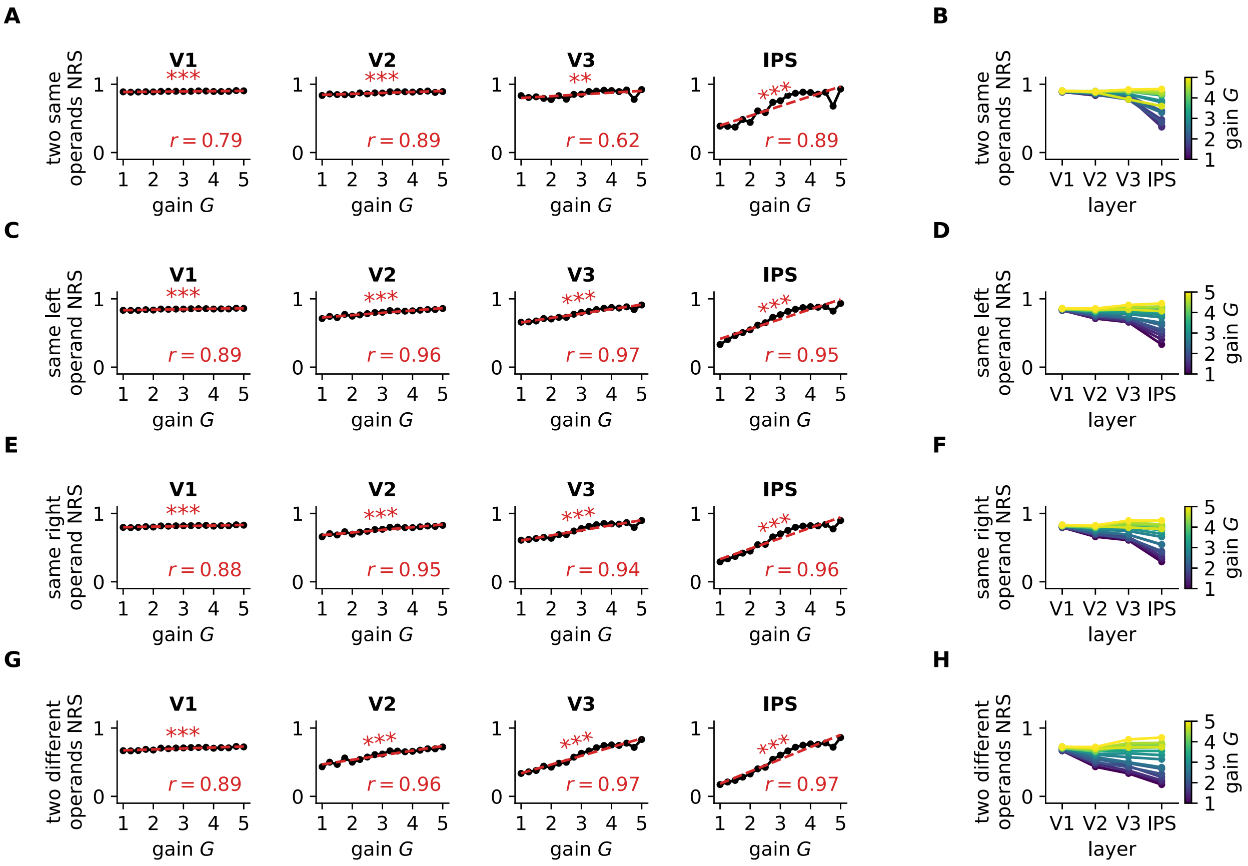

Fig. S2. Impact of hyper-excitability on two same operands NRS, left same operand NRS, right same operand NRS, and two different operands NRS.

A-B. Relationship between pDNN NRS and neural gain G for NRS between pairs of problems that share two operands, with linear regression lines (red) showing the strength and direction of the correlation. Summary showing the evolution of NRS measures across model layers (V1→IPS) for different levels of neural gain G, depicted as a color gradient from blue ($G = 1$) to yellow ($G = 5$). NRS between pairs of problems that share two operands increases with neural gain across all layers, indicating reduced differentiation between related problems. **C-F.** NRS between pairs of problems that share their left (C-D) or right (E-F) operand increases with neural gain across all layers, suggesting reduced differentiation between problems that share one of their operands. **G-H.** NRS between pairs of problems that do not share any operand also increases with neural gain across all layers, suggesting a global reduced differentiation between problems. ** $p < 0.01$, *** $p < 0.001$.

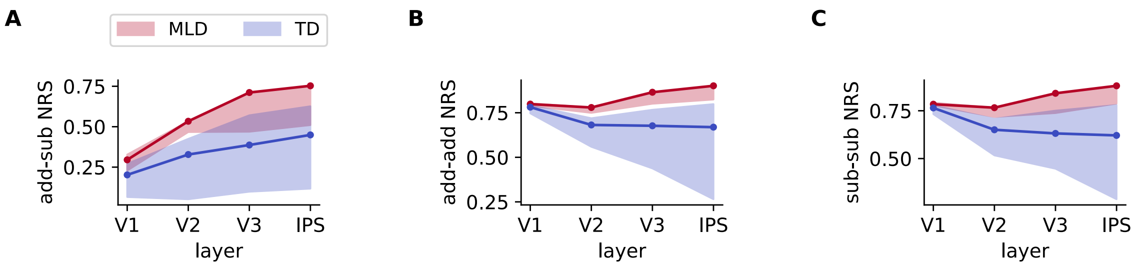

Fig. S3. pDNNs predict deficit in neural differentiation between and within operation. Comparison of NRS across pDNN layers (V1 to IPS) for models matched to children with MLD (red) and typically developing (TD) children (blue). MLD pDNNs show significantly higher **A.** add-sub NRS, **B.** add-add NRS, and **C.** sub-sub NRS compared to TD pDNNs across all layers, indicating both reduced neural differentiation between problem types and within problem types. The effect size of the difference in NRS between MLD and TD pDNNs increases along the network hierarchy, suggesting a more pronounced deficit in higher-order processing regions.

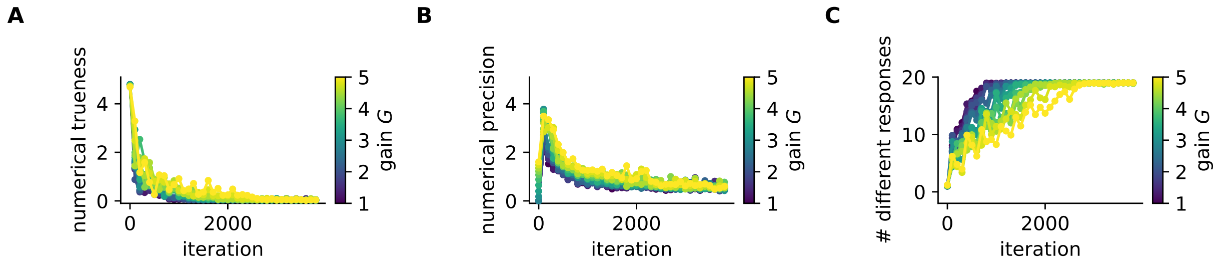

Fig. S4. Early deficits in systematic numerical error and numerical precision are overcome with additional training.

Evolution of DNN numerical properties for different levels of neural excitability measured as neural gain *G*. Neural gain *G* is represented by color, varying from blue ($G = 1$) to yellow ($G = 5$). **A-C.** Early in training, as neural excitability increases, **A.** systematic numerical error and **B.** numerical imprecision increase, and **C.** the number of different responses used decrease. All these deficits are overcome with more training, i.e. **A.** systematic numerical trueness, **B.** numerical imprecision, and **C.** number of different responses used reach similar higher levels with additional training regardless of neural excitability.

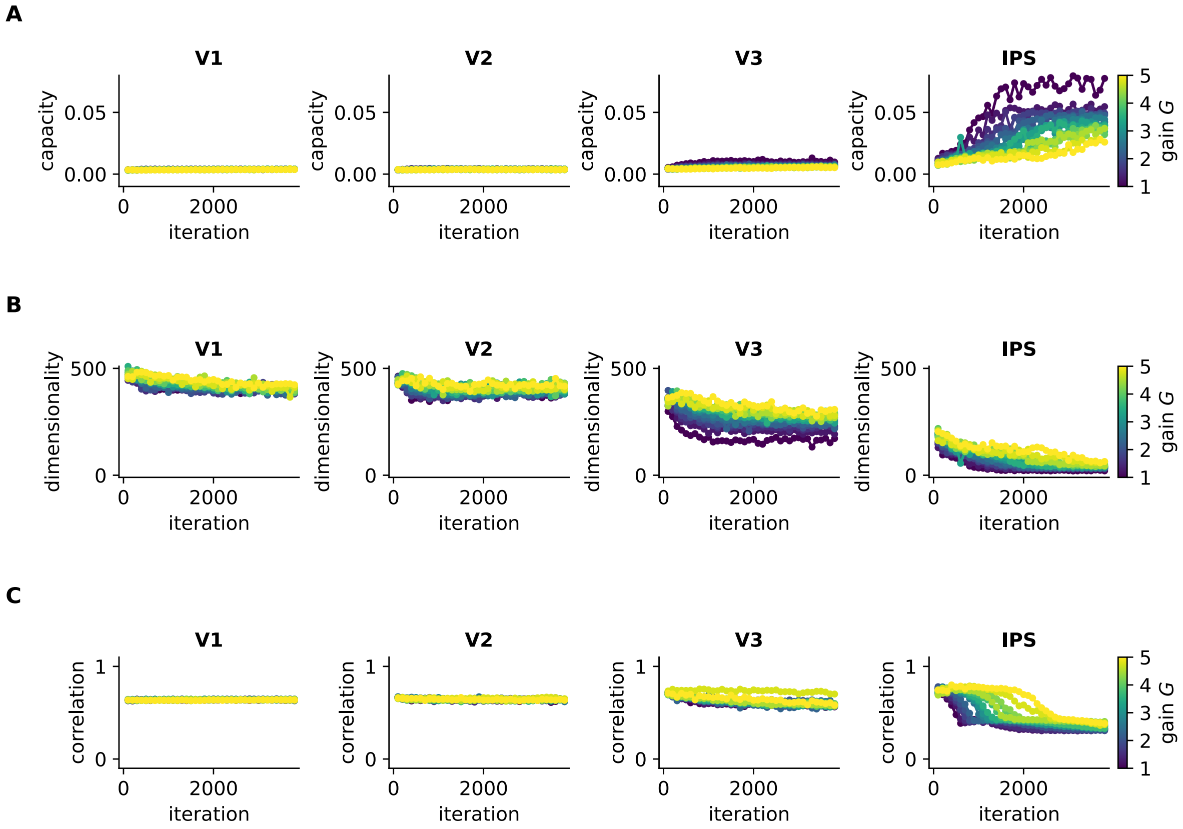

Fig. S5. Degradation of manifold geometrical properties of neural representations caused by hyper-excitability are partially overcome with more training.

Evolution of manifold geometric properties for different levels of neural excitability measured as neural gain *G*. Neural gain *G* is represented by color, varying from blue (*G* = 1) to yellow (*G* = 5). **A-C.** As neural excitability increases, **A.** manifold capacity decreases, **B.** manifold dimensionality increases **C.** and correlation between center of manifolds increases, indicating harder to discrimate, more complex and more aligned representations. Not all of these deficits are overcome even with additional training.

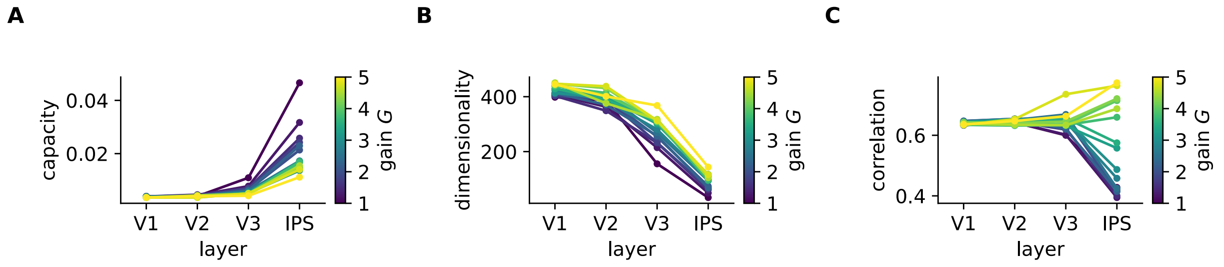

Fig. S6. Neural hyper-excitability degrades manifold geometry of latent representations across layers in pDNNs**.**

A-C. Three key manifold properties in the IPS layer of pDNNs change with neural gain levels across layers. **A.** Manifold capacity, reflecting the separability of neural representations, shows decrease with higher excitation and increase along hierarchy, indicating that hyper-excitability makes it more difficult to distinguish between different numerical manifolds and that it is easier to distinguish between different numerical manifolds higher in the hierarchy. **B.** Manifold dimensionality, indicating the complexity of the representational space, increases with greater neural gain and decreases along hierarchy, suggesting that hyper-excitability leads to more complex and less efficiently organized representations, and that representation are simplified along the hierarchy. **C.** Correlations between manifold centers, relating to the alignment of representations, increase with neural gain and either decrease along hierarchy for smaller gain or increase along hierarchy for higher gain, implying that hyper-excitability causes the centers of different numerical manifolds to become more aligned, potentially leading to increased interference between representations, and that this alignement is amplified along hierarchy for higher gains but reduced along the hierarchy for smaller gains.

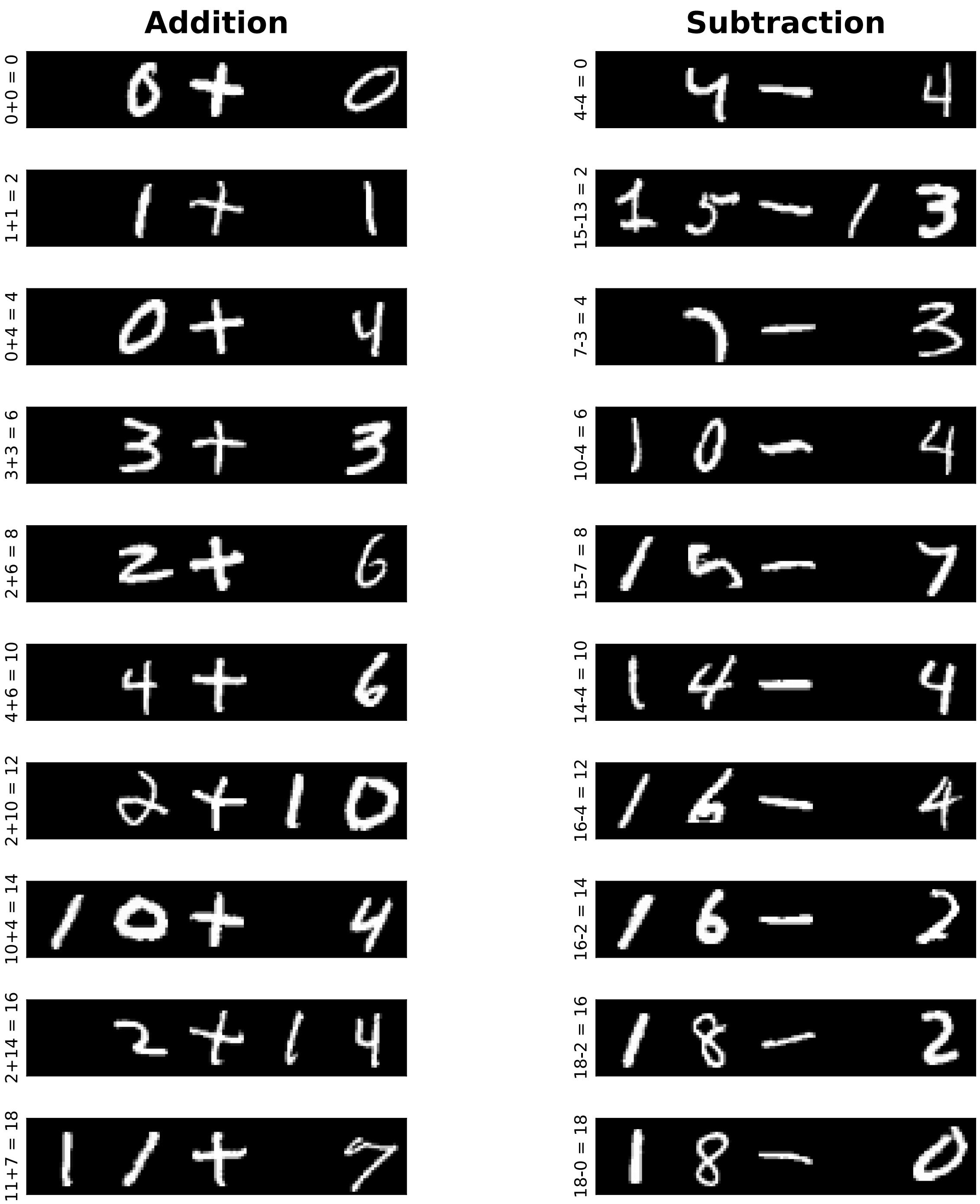

Fig. S7. Examples of stimuli of addition and subtraction (Handwritten).

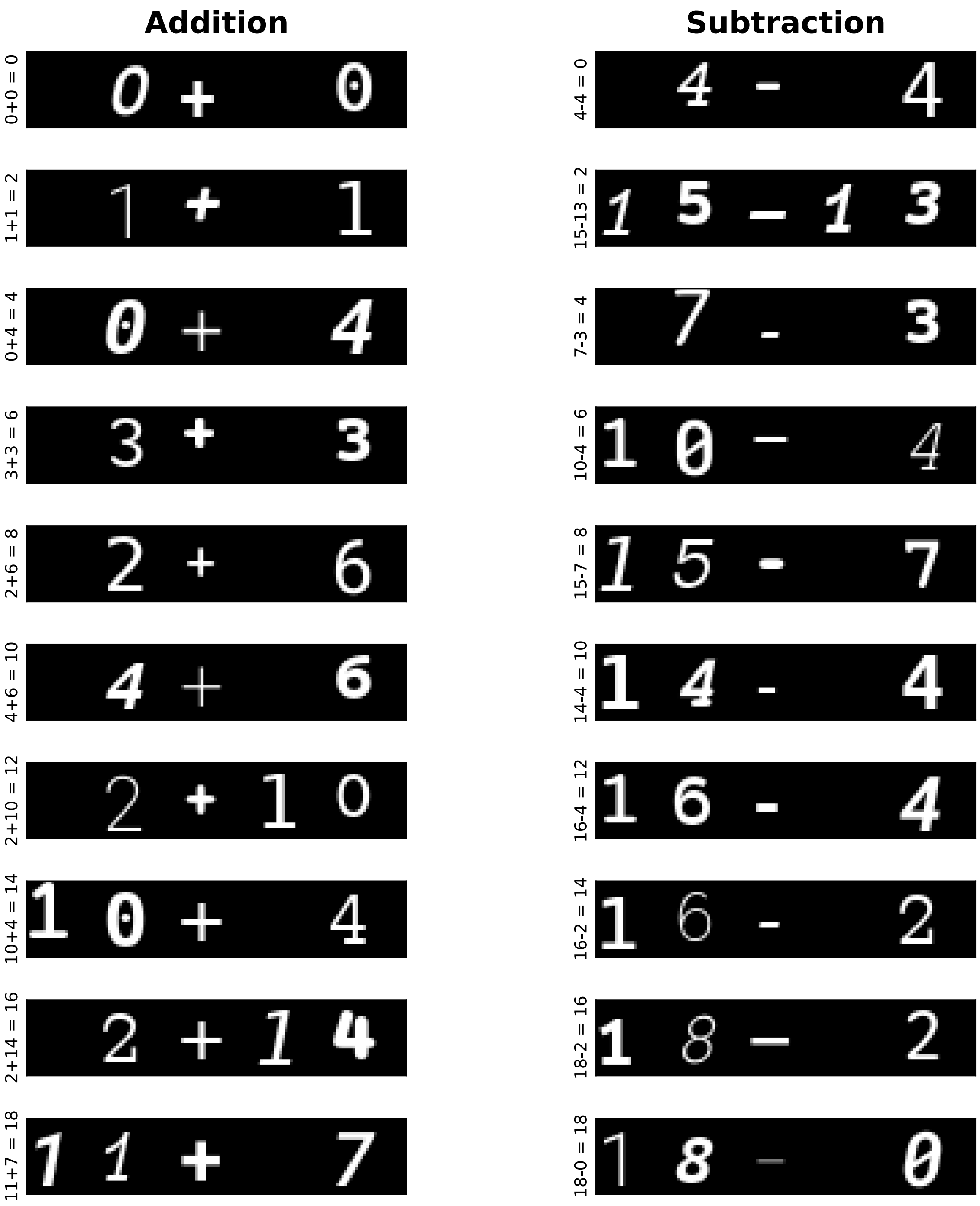

Fig. S8. Examples of stimuli of addition and subtraction (Printed font).

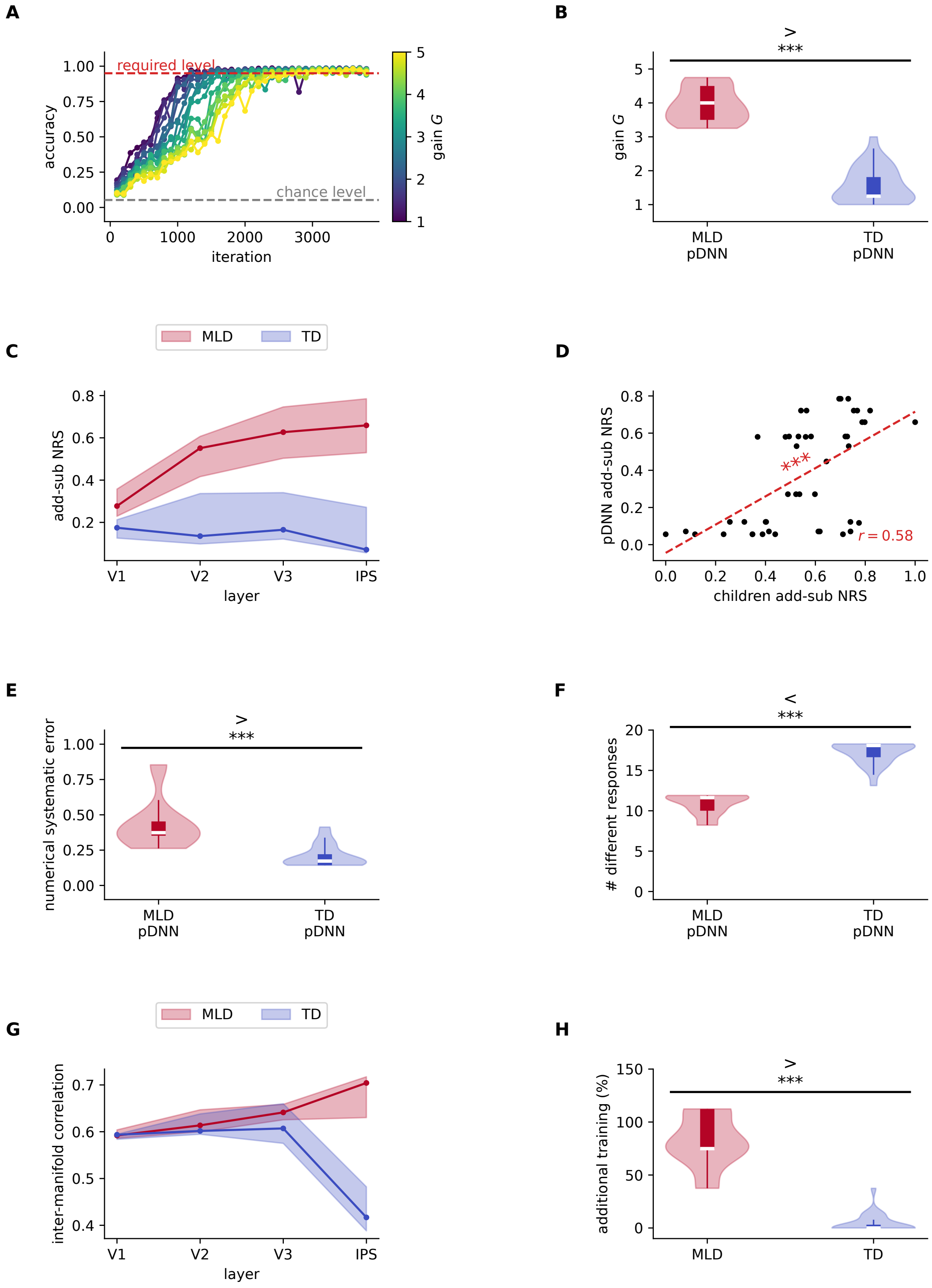

A
B
C
D
E
F
G
H
required level
chance level
accuracy
iteration
gain G
MLD
TD
pDNN
***
add-sub NRS
layer
V1
V2
V3
IPS
pDNN add-sub NRS
children add-sub NRS
r = 0.58
numerical systematic error
different responses
inter-manifold correlation
additional training (%)

Fig. S9. Replication of main results when stimuli include both handwritten stimuli and stimuli generated using printed fonts.

A. Figure 2A. **B.** Figure 3C. **C.** Figure 5A. **D.** Figure 5B. **E.** Figure 6E. **F.** Figure 6G. **G.** Figure 7F. **H.** Figure 8B.

	TD children (12F/12M)	MLD children (13F/8M)
**Age**	8.40±0.64	8.34±0.65
**Full-scale IQ (WASI)**	111.83±10.26	108.33±10.69
**Reading Comprehension (WIAT II)**	109.04±10.19	104.10±9.45
**Numerical Operation (WIAT II)**	110.75±9.60	85.00±4.29

Table S1. Cognitive profiles of participants.
Refer to *(20)* Table 1 for a more complete description of the cognitive profiles.

REFERENCES AND NOTES

1. F. A. Campbell, E. P. Pungello, S. Miller-Johnson, M. Burchinal, C. T. Ramey, The development of cognitive and academic abilities: Growth curves from an early childhood educational experiment. *Dev. Psychol.* **37**, 231–242 (2001).

2. I. Xenidou-Dervou, J. E. H. Van Luit, E. H. Kroesbergen, I. Friso-van den Bos, L. M. Jonkman, M. van der Schoot, E. C. M. Van Lieshout, Cognitive predictors of children's development in mathematics achievement: A latent growth modeling approach. *Dev. Sci.* **21**, e12671 (2018).

3. M. S. Thomas, D. Annaz, D. Ansari, G. Scerif, C. Jarrold, A. Karmiloff-Smith, Using developmental trajectories to understand developmental disorders. *J. Speech Lang. Hear. Res.* **52**, 336–358 (2009).

4. S. S. Wu, L. Chen, C. Battista, A. K. Smith Watts, E. G. Willcutt, V. Menon, Distinct influences of affective and cognitive factors on children's non-verbal and verbal mathematical abilities. *Cognition* **166**, 118–129 (2017).

5. D. C. Geary, Consequences, characteristics, and causes of mathematical learning disabilities and persistent low achievement in mathematics. *J. Dev. Behav. Pediatr.* **32**, 250–263 (2011).

6. W. Fias, V. Mellon, D. Szucs, Multiple components of developmental dyscalculia. *Trends Neurosci. Educ.* **2**, 43–47 (2013).

7. L. S. Fuchs, D. L. Compton, D. Fuchs, K. Paulsen, J. D. Bryant, C. L. Hamlett, The prevention, identification, and cognitive determinants of math difficulty. *J. Educ. Psychol.* **97**, 493–513 (2005).

8. C. Koedel, E. Tyhurst, Math skills and labor-market outcomes: Evidence from a resume-based field experiment. *Econ. Educ. Rev.* **31**, 131–140 (2012).

9. F. L. Riverabatiz, Quantitative literacy and the likelihood of employment among young adults in the United States. *J. Hum. Resour.* **27**, 313–328 (1992).

10. H. Xiong, C. Chu, L. Fan, M. Song, J. Zhang, Y. Ma, R. Zheng, J. Zhang, Z. Yang, T. Jiang, The digital twin brain: A bridge between biological and artificial intelligence. *Intel. Comput.* **2**, 0055 (2023).

11. I. Ustyuzhaninov, M. F. Burg, S. A. Cadena, J. Fu, T. Muhammad, K. Ponder, E. Froudarakis, Z. Ding, M. Bethge, A. S. Tolias, Digital twin reveals combinatorial code of non-linear computations in the mouse primary visual cortex. bioRxiv, 2022.2002.2010.479884 [Preprint] (2022), https://doi.org/10.1101/2022.02.10.479884.

12. D. Jones, C. Snider, A. Nassehi, J. Yon, B. Hicks, Characterising the digital twin: A systematic literature review. *CIRP J. Manuf. Sci. Technol.* **29**, 36–52 (2020).

13. P. K. Mistry, A. Strock, R. Liu, G. Young, V. Menon, Learning-induced reorganization of number neurons and emergence of numerical representations in a biologically inspired neural network. *Nat. Commun.* **14**, 3843 (2023).

14. J. Kubilius, M. Schrimpf, A. Nayebi, D. Bear, D. L. Yamins, J. J. DiCarlo, CORnet: Modeling the neural mechanisms of core object recognition. BioRxiv 408385 [Preprint] (2018), https://doi.org/10.1101/408385.

15. H. Chang, V. Menon, Theories of dyscalculia in *The Cambridge Handbook of Dyslexia and Dyscalculia*, M. A. Skeide, Ed. (Cambridge Univ. Press, 2022).

16. V. Menon, H. Chang, Emerging neurodevelopmental perspectives on mathematical learning. *Dev. Rev.* **60**, 100964 (2021).

17. M. Rosenberg-Lee, S. Ashkenazi, T. W. Chen, C. B. Young, D. C. Geary, V. Menon, Brain hyper-connectivity and operation-specific deficits during arithmetic problem solving in children with developmental dyscalculia. *Dev. Sci.* **18**, 351–372 (2015).

18. D. C. Geary, M. K. Hoard, J. Byrd-Craven, L. Nugent, C. Numtee, Cognitive mechanisms underlying achievement deficits in children with mathematical learning disability. *Child Dev.* **78**, 1343–1359 (2007).

19. S. A. Ostad, Developmental progression of subtraction strategies: A comparison of mathematically normal and mathematically disabled children. *Eur. J. Spec. Needs Educ.* **14**, 21–36 (1999).

20. L. Chen, T. Iuculano, P. Mistry, J. Nicholas, Y. Zhang, V. Menon, Linear and nonlinear profiles of weak behavioral and neural differentiation between numerical operations in children with math learning difficulties. *Neuropsychologia* **160**, 107977 (2021).

21. B. De Smedt, I. D. Holloway, D. Ansari, Effects of problem size and arithmetic operation on brain activation during calculation in children with varying levels of arithmetical fluency. *Neuroimage* **57**, 771–781 (2011).

22. V. Menon, Arithmetic in the child and adult brain in *The Oxford Handbook of Numerical Cognition*, R. Cohen Kadosh, A. Dowker, Eds. (Oxford Univ. Press, 2014).

23. V. Menon, A. Padmanabhan, F. Schwartz, Cognitive neuroscience of dyscalculia and math learning disabilities in *Oxford Handbook of Developmental Cognitive Neuroscience*, (Oxfor Univ. Press, 2020).

24. L. Peters, B. De Smedt, Arithmetic in the developing brain: A review of brain imaging studies. *Dev. Cogn. Neurosci.* **30**, 265–279 (2018).

25. G. R. Price, M. M. Mazzocco, D. Ansari, Why mental arithmetic counts: Brain activation during single digit arithmetic predicts high school math scores. *J. Neurosci.* **33**, 156–163 (2013).

26. A. Faye, S. Jacquin-Courtois, E. Reynaud, M. Lesourd, J. Besnard, F. Osiurak, Numerical cognition: A meta-analysis of neuroimaging, transcranial magnetic stimulation and brain-damaged patients studies. *Neuroimage Clin.* **24**, 102053 (2019).

27. J. Tablante, L. Krossa, T. Azimi, L. Chen, Dysfunctions associated with the intraparietal sulcus and a distributed network in individuals with math learning difficulties: An ALE meta-analysis. *Hum. Brain Mapp.* **44**, 2726–2740 (2023).

28. M. Arsalidou, M. Pawliw-Levac, M. Sadeghi, J. Pascual-Leone, Brain areas associated with numbers and calculations in children: Meta-analyses of fMRI studies. *Dev. Cogn. Neurosci.* **30**, 239–250 (2018).

29. M. Arsalidou, M. J. Taylor, Is 2+2=4? Meta-analyses of brain areas needed for numbers and calculations. *Neuroimage* **54**, 2382–2393 (2011).

30. S. Ashkenazi, M. Rosenberg-Lee, A. W. Metcalfe, A. G. Swigart, V. Menon, Visuo–spatial working memory is an important source of domain-general vulnerability in the development of arithmetic cognition. *Neuropsychologia* **51**, 2305–2317 (2013).

31. S. Ashkenazi, M. Rosenberg-Lee, C. Tenison, V. Menon, Weak task-related modulation and stimulus representations during arithmetic problem solving in children with developmental dyscalculia. *Dev. Cogn. Neurosci.* **2**, S152–S166 (2012).

32. J. Bulthé, J. Prinsen, J. Vanderauwera, S. Duyck, N. Daniels, C. R. Gillebert, D. Mantini, H. P. O. de Beeck, B. De Smedt, Multi-method brain imaging reveals impaired representations of number as well as altered connectivity in adults with dyscalculia. *Neuroimage* **190**, 289–302 (2019).

33. D. Jolles, S. Ashkenazi, J. Kochalka, T. Evans, J. Richardson, M. Rosenberg-Lee, H. Zhao, K. Supekar, T. Chen, V. Menon, Parietal hyper-connectivity, aberrant brain organization, and circuit-based biomarkers in children with mathematical disabilities. *Dev. Sci.* **19**, 613–631 (2016).

34. R. A. Abreu-Mendoza, M. Pincus, Y. Chamorro, D. Jolles, E. Matute, M. Rosenberg-Lee, Parietal and hippocampal hyper-connectivity is associated with low math achievement in adolescence–A preliminary study. *Dev. Sci.* **25**, e13187 (2022).

35. G. Zacharopoulos, F. Sella, R. Cohen Kadosh, The impact of a lack of mathematical education on brain development and future attainment. *Proc. Natl. Acad. Sci. U.S.A.* **118**, e2013155118 (2021).

36. G. Zacharopoulos, F. Sella, U. Emir, R. C. Kadosh, The relation between parietal GABA concentration and numerical skills. *Sci. Rep.* **11**, 17656 (2021).

37. B. Krause, C. Y. Looi, M. Dresler, R. C. Kadosh, The neurochemistry of mathematical genius: Reduced frontal excitation/inhibition balance in an expert calculator. *Neuroscience* **392**, 252–257 (2018).

38. G. Zacharopoulos, F. Sella, K. C. Kadosh, C. Hartwright, U. Emir, R. C. Kadosh, Predicting learning and achievement using GABA and glutamate concentrations in human development. *PLOS Biol.* **19**, e3001325 (2021).

39. G. Zacharopoulos, F. Sella, K. Cohen Kadosh, U. Emir, R. Cohen Kadosh, The effect of parietal glutamate/GABA balance on test anxiety levels in early childhood in a cross-sectional and longitudinal study. *Cereb. Cortex* **32**, 3243–3253 (2022).

40. N. van Bueren, S. van der Ven, F. Sella, R. C. Kadosh, The role of Excitation/Inhibition ratio (E/I) in cognitive learning and using neurostimulation to alter E/I and improve cognition. *Brain Stimul.* **16**, 170 (2023).

41. N. E. Van Bueren, S. H. G. Van Der Ven, S. Hochman, F. Sella, R. C. Kadosh, Explaining and predicting the effects of neurostimulation via neuronal excitation/inhibition on learning. bioRxiv 2022.04.29.489988 [Preprint] (2022), https://doi.org/10.1101/2022.04.29.489988.

42. N. E. R. van Bueren, S. H. G. van der Ven, S. Hochman, F. Sella, R. Cohen Kadosh, Human neuronal excitation/inhibition balance explains and predicts neurostimulation induced learning benefits. *PLOS Biol.* **21**, e3002193 (2023).

43. G. Kim, J. Jang, S. Baek, M. Song, S. B. Paik, Visual number sense in untrained deep neural networks. *Sci. Adv.* **7**, eabd6127 (2021).

44. K. Nasr, P. Viswanathan, A. Nieder, Number detectors spontaneously emerge in a deep neural network designed for visual object recognition. *Sci. Adv.* **5**, eaav7903 (2019).

45. J. D. Zevin, M. S. Seidenberg, Simulating consistency effects and individual differences in nonword naming: A comparison of current models. *J. Mem. Lang.* **54**, 145–160 (2006).

46. M. A. L. Ralph, C. Lowe, T. T. Rogers, Neural basis of category-specific semantic deficits for living things: Evidence from semantic dementia, HSVE and a neural network model. *Brain* **130**, 1127–1137 (2007).

47. T. T. Rogers, M. A. L. Ralph, P. Garrard, S. Bozeat, J. L. McClelland, J. R. Hodges, K. Patterson, Structure and deterioration of semantic memory: A neuropsychological and computational investigation. *Psychol. Rev.* **111**, 205–235 (2004).

48. M. C. MacDonald, M. H. Christiansen, Reassessing working memory: Comment on Just and Carpenter (1992) and Waters and Caplan (1996). *Psychol. Rev.* **109**, 35–54 (2002).

49. T. Iuculano, Neurocognitive accounts of developmental dyscalculia and its remediation. *Prog. Brain Res.* **227**, 305–333 (2016).

50. P. Barrouillet, M. Mignon, C. Thevenot, Strategies in subtraction problem solving in children. *J. Exp. Child Psychol.* **99**, 233–251 (2008).

51. A. Bhatia, S. Moza, U. S. Bhalla, Precise excitation-inhibition balance controls gain and timing in the hippocampus. *eLife* **8**, e43415 (2019).

52. H. Idei, S. Murata, Y. Yamashita, T. Ogata, Homogeneous intrinsic neuronal excitability induces overfitting to sensory noise: A robot model of neurodevelopmental disorder. *Front. Psych.* **11**, 762 (2020).

53. P. Trapp, R. Echeveste, C. Gros, E-I balance emerges naturally from continuous Hebbian learning in autonomous neural networks. *Sci. Rep.* **8**, 8939 (2018).

54. S. Zhou, Y. Yu, Synaptic E-I balance underlies efficient neural coding. *Front. Neurosci.* **12**, 46 (2018).

55. K. Cohen Kadosh, B. Krause, A. J. King, J. Near, R. Cohen Kadosh, Linking GABA and glutamate levels to cognitive skill acquisition during development. *Hum. Brain Mapp.* **36**, 4334–4345 (2015).

56. S. Chung, D. D. Lee, H. Sompolinsky, Classification and geometry of general perceptual manifolds. *Phys. Rev. X* **8**, 031003 (2018).

57. U. Cohen, S. Chung, D. D. Lee, H. Sompolinsky, Separability and geometry of object manifolds in deep neural networks. *Nat. Commun.* **11**, 746 (2020).

58. D. Wechsler, 1992 Wechsler Individual Achievement Test.

59. Accuracy (trueness and precision) of measurement methods and results (ISO 5725-1:2023) International Organization for Standardization Geneva 2023.

60. J. Campbell, Mechanisms of simple addition and multiplication: A modified network-interference theory and simulation. *Math. Cognit.* **1**, 121–164 (1995).

61. J. I. Campbell, J. C. Timm, Adults' strategy choices for simple addition: Effects of retrieval interference. *Psychon. Bull. Rev.* **7**, 692–699 (2000).

62. R. S. Siegler, Strategy choice procedures and the development of multiplication skill. *J. Exp. Psychol. Gen.* **117**, 258–275 (1988).

63. S. Chung, D. D. Lee, H. Sompolinsky, Linear readout of object manifolds. *Phys. Rev. E* **93**, 060301 (2016).

64. T. Iuculano, M. Rosenberg-Lee, J. Richardson, C. Tenison, L. Fuchs, K. Supekar, V. Menon, Cognitive tutoring induces widespread neuroplasticity and remediates brain function in children with mathematical learning disabilities. *Nat. Commun.* **6**, 8453 (2015).

65. M. Amalric, S. Dehaene, Origins of the brain networks for advanced mathematics in expert mathematicians. *Proc. Natl. Acad. Sci. U.S.A.* **113**, 4909–4917 (2016).

66. M. Amalric, S. Dehaene, Cortical circuits for mathematical knowledge: Evidence for a major subdivision within the brain's semantic networks. *Philos. Trans. R. Soc. B Biol. Sci.* **373**, 20160515 (2018).

67. M. Grotheer, B. Jeska, K. Grill-Spector, A preference for mathematical processing outweighs the selectivity for Arabic numbers in the inferior temporal gyrus. *Neuroimage* **175**, 188–200 (2018).

68. P. Pinheiro-Chagas, A. Daitch, J. Parvizi, S. Dehaene, Brain mechanisms of arithmetic: A crucial role for ventral temporal cortex. *J. Cogn. Neurosci.* **30**, 1757–1772 (2018).

69. D. J. Yeo, C. Pollack, R. Merkley, D. Ansari, G. R. Price, The "Inferior Temporal Numeral Area" distinguishes numerals from other character categories during passive viewing: A representational similarity analysis. *Neuroimage* **214**, 116716 (2020).

70. R. M. French, Catastrophic forgetting in connectionist networks. *Trends Cogn. Sci.* **3**, 128–135 (1999).

71. M. McCloskey, N. J. Cohen. Catastrophic interference in connectionist networks: The sequential learning problem in *Psychology of Learning and Motivation*. (Elsevier, vol. 24, 1989), pp. 109–165.

72. L. Deng, The mnist database of handwritten digit images for machine learning research [best of the web]. *IEEE Signal Process. Mag.* **29**, 141–142 (2012).

73. D. P. Kingma, J. Ba, Adam: A method for stochastic optimization. arXiv:1412.6980 [cs.LG] (2014), https://doi.org/10.48550/arXiv.1412.6980.

74. K. He, X. Zhang, S. Ren, J. Sun, in *Proceedings of the IEEE Conference on Computer Vision and Pattern Recognition*. 2016, pp. 770–778.
